# Investigation on the Model-Based Control Performance in Vehicle Safety Critical Scenarios with Varying Tyre Limits

**DOI:** 10.3390/s21165372

**Published:** 2021-08-09

**Authors:** Aleksandr Sakhnevych, Vincenzo Maria Arricale, Mattia Bruschetta, Andrea Censi, Enrico Mion, Enrico Picotti, Emilio Frazzoli

**Affiliations:** 1 Department of Industrial Engineering, University of Napoli Federico II, 80125 Naples, Italy; vincenzomaria.arricale@unina.it; 2 Department of Information Engineering, University of Padova, Via Gradenigo, 6/B, 35131 Padova, Italy; mattia.bruschetta@dei.unipd.it (M.B.); picottie@dei.unipd.it (E.P.); 3 Institute for Dynamical Systems and Control, ETH Zurich, 8092 Zurich, Switzerland; acensi@idsc.mavt.ethz.ch (A.C.); enmion@ethz.ch (E.M.); efrazzoli@ethz.ch (E.F.)

**Keywords:** model-based control, vehicle dynamic potential, tyre thermodynamics, tyre wear, weather influence, vehicle safety, double lane change, safety optimization

## Abstract

In recent years the increasing needs of reducing the costs of car development expressed by the automotive market have determined a rapid development of virtual driver prototyping tools that aims at reproducing vehicle behaviors. Nevertheless, these advanced tools are still not designed to exploit the entire vehicle dynamics potential, preferring to assure the minimum requirements in the worst possible operating conditions instead. Furthermore, their calibration is typically performed in a pre-defined strict range of operating conditions, established by specific regulations or OEM routines. For this reason, their performance can considerably decrease in particularly crucial safetycritical situations, where the environmental conditions (rain, snow, ice), the road singularities (oil stains, puddles, holes), and the tyre thermal and ageing phenomena can deeply affect the adherence potential. The objective of the work is to investigate the possibility of the physical model-based control to take into account the variations in terms of the dynamic behavior of the systems and of the boundary conditions. Different scenarios with specific tyre thermal and wear conditions have been tested on diverse road surfaces validating the designed model predictive control algorithm in a hardware-in-the-loop real-time environment and demonstrating the augmented reliability of an advanced virtual driver aware of available information concerning the tyre dynamic limits. The multidisciplinary proposal will provide a paradigm shift in the development of strategies and a solid breakthrough towards enhanced development of the driving automatization systems, unleashing the potential of physical modeling to the next level of vehicle control, able to exploit and to take into account the multi-physical tyre variations.

## 1. Introduction

The information concerning the vehicle’s non-linear physical limits depending on the thermal and wear states of tyres, the pavement characteristics, and the boundary conditions (wet or icy ground, under-inflated or worn tyre, etc.) represents a fundamental additional value for the optimal behavior of safety- and performance-oriented control logics [1,2,3].

Virtual driver prototyping is becoming an increasingly exploited tool, allowing the car manufacturer to perform the majority of the testing campaign already in the design phase of the vehicle. Specific prototyping choices can be reproduced and evaluated in any condition within the virtual environment, also at the limit of performance, minimizing the time-to-market and connected costs [4,5].

In this field, closed-loop control strategies have been widely studied in past years to address the problem of path following for autonomous driving cars. Examples can be found in [6], where a nested PID steering control has been designed for the lane-keeping task, and more recently in [7], where a pure pursuit controller has been specifically developed for path tracking. The most recent VD implementations rely on a vehicle controller based on a non-linear model predictive control (NMPC) technique, which is a model-based control strategy able to compute the optimal sequence of control inputs over a prediction horizon, by minimizing a tailored cost function [8,9]. The control technique is applied in a receding horizon mode and is capable of handling constraints and the intrinsic non-linearities of the vehicle model [10].

The main advantages of the NMPC approach are the capability of the controller of handling all significant features of the process dynamics directly: in this way, the constraints on variables involved in the task (track limits, actuator constraints) can be easily integrated into the optimal control problem, hence guaranteeing the maximal exploitation of vehicle capabilities. Moreover, it is a predictive technique that allows optimizing the vehicle behavior over a future horizon in time, and therein system states and controls. In this way, the controller is allowed to retrieve information about future vehicle behavior and about possible dangerous situations, aiming at anticipating actions and providing suitable controls for challenging vehicle handling.

The objective of the work consists in the integration of the information concerning the tyre dynamic limits within the definition of a virtual driver (VD), implemented as a vehicle controller aiming at testing the vehicle behavior at limit of handling condition, and demonstrating the advantages in terms of both enhanced active safety and optimized performance. An interesting VD definition that addresses the problem of real-time obstacle avoidance on low-friction road surfaces has been proposed in [11], where the code generation tool ACADO [12] has been used to define and solve the NMPC problem. Another similar implementation of such a controller for an autonomous ground vehicle has been proposed in [13], where the controller has been also validated in co-simulation with a hard real-time dSPACE DS1005 Autobox system. The vehicle model employed in the both implementations has consisted of a four-wheel vehicle, where tyres have been described by means of a linear tyre model and Fiala tyre model for longitudinal and lateral dynamics, respectively [14,15]. The inputs are the steering angle and the front/rear braking ratios, while the bounds are defined through specifically defined spatial constraints. A different virtual driver definition, especially designed for high performance vehicles, has been developed in [16]: here, the vehicle model integrates longitudinal load transfer and Pacejka’s lateral tyres forces model. The controller implementation has been tested in a real-time co-simulation with a commercial software VI-CarRealTime (VI-CRT) within a double lane change (DLC) maneuver, where the abilities of the controller have been demonstrated with high speed operating conditions and a challenging track geometry. The NMPC strategy has also been applied in racing environment as the autonomous vehicle controller for handling 1:43 scale, RC electric vehicles [17] and autonomous racecar [18], with the specific purpose of achieving aggressive maneuvering and lap time minimization.

In this work, the authors aim to investigate the possibility to employ the model-based strategies to control the non-linear time-dependent system, i.e., the full vehicle model with temperature and wear sensitive tyres operating in completely different environmental conditions. To perform the study, the standardized DLC maneuver, currently employed for the validation of virtual driver and advanced driving assistance systems (ADAS) [16,19,20], is implemented in Matlab/Simulink virtual environment. The vehicle and tyre models have been characterized and validated for a reference GT vehicle, identifying the requisite complex tyre–road coupled phenomena concerning the temperature, wear, and road pavement dependencies [21,22]. Four different roads, i.e., dry, wet, snowy, and icy, two diverse tyre mileages, i.e., new and worn, and three thermal tyre conditions are combined and analyzed in the study to understand which could be the advantages of the employment of the model-based controllers, aware of the tyre instantaneous characteristics, boundary operating and weather conditions, and overall vehicle dynamic potential [23]. The model-based control logics, able to make use of the additional information concerning the dynamic limits of the system, is tested in co-simulation with a 14 degrees-of-freedom vehicle plant model, where the tyres are described by means of a Pacejka’s magic formula (MF) model. The vehicle controller is based on a robust and computationally effective non-linear model-predictive-control (NMPC), implemented in the open-source NMPC software MATMPC [24], able to take into account the additional instantaneous information concerning the varying adherence potential and the vehicle non-linearities. The information concerning the vehicle non-linear physical limits depending on the thermal and wear states of tyres, the pavement characteristics and the boundary conditions (wet or icy ground, under-inflated or worn tyre, etc.) represents a fundamental additional value for the optimal behavior of safety- and performance-oriented control logics [25,26,27], as it allows to maximize the potential to avoid obstacles and to reduce the severity of collisions [28].

The authors aim to lay the foundation of the future advanced driving systems, sensitive to environmental conditions and adaptive to continuously varying characteristics of the underlying non-linear system. Being currently mainly based on mere empirical calibration, the physical model-based estimation can represent a crucial factor towards the improvement of the pedestrians’ and passengers’ active safety, enabling the management of the activation threshold ranges on the basis of the instantaneous operating and the environmental boundary conditions [29,30]. This can be already employed in the current ADAS to communicate to the driver the necessity to co-act in specific situations, but it also constitutes a fundamental root for the future driving automatization [31,32].

The paper is organized as follows: Section 2 introduces the problem description concerning complex phenomena linked with the tyre–road interaction and their influence on the overall vehicle dynamics; Section 3 describes the advanced methodologies developed to characterize, model, and reproduce the dynamic behavior of the real system in the virtual environment, and introduces the adopted model-based control, evidencing the peculiarities of the designed cost function; in Section 4 the outputs of the conducted simulations employing different road surfaces, in adverse boundary conditions and with diverse states of the tyres are discussed, addressing particular attention towards the control strategies. Finally, in Section 5, a discussion on the next developments and the conclusions are presented.

## 2. Problem Description

A proper understanding of the tyre dynamic behavior and of its multiple intrinsic dependencies is a crucial topic for tyre manufacturers, to improve tyre performance and durability, for users, to set the optimal working conditions, and for researchers, to develop computationally efficient mathematical models able to represent the experimental behavior with a high degree of accuracy. Friction phenomenon, arising at the tyre–road interface, originates from three physical contributions: the adhesive term relative to molecular Van der Waals links arising between the two counter surfaces in mutual contact, the hysteretic term linked to the deformation losses within the elastomeric material, and the wear term [33,34]. All of them are deeply interconnected and dependent on the specific tyre working conditions, in terms of sliding velocity, temperature, and pressure distributions, arising at the tyre contact patch as a result of different excitation spatial frequency spectra, representative of diverse types of road pavement [35]. Furthermore, tyres may deeply modify their dynamic behavior over time due to ageing effects, influencing the dynamic potential of the overall vehicle [22].

**Figure 1:**
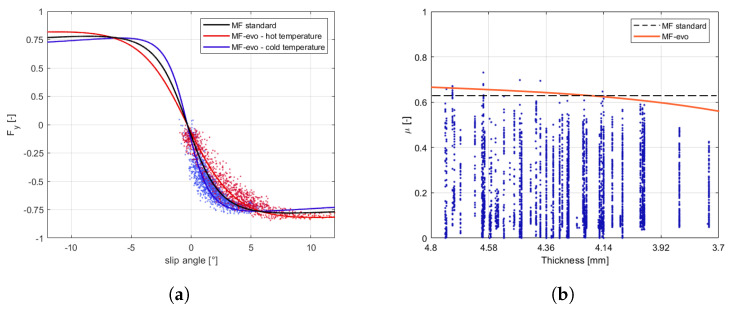
Tyre behavior variations. (**a**) Compound temperature influence on the characteristic interaction shape. (**b**) Wear effect on available grip.

**Figure 2:**
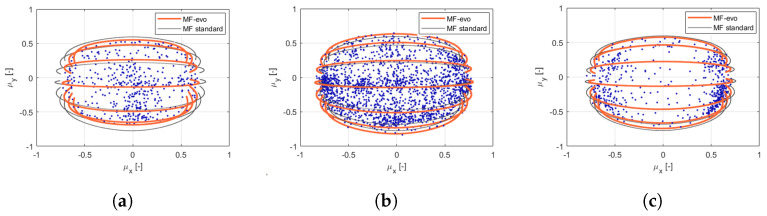
MF-based standard and evo tyre models compared with the experimental points in three thermal ranges: under-heating condition (**a**), optimal temperature (**b**), over-heating condition (**c**), (camber angle = −2deg|vertical load = 3000N).

The enrichment of the vehicle state with the information concerning the tyre instantaneous and potential friction will allow, taking into account the tyre multi-physical variations (Figure 1 and Figure 2), represents a key point in the development of control logics, able to adapt to sudden variation in boundary conditions in order to guarantee the vehicles higher stability in critical scenarios. Indeed, in the Figure 2 it is possible to observe how the adherence ellipse changes in three tyre thermal ranges.

The aim of the proposed adaptive control will be to avoid collisions and to minimize risks in any environmental condition, validating all the scenarios on interest in a highly accurate simulation environment. The information concerning the vehicle non-linear physical limits depending on the thermal and wear states of tyres, the pavement characteristics and the boundary conditions (wet or icy ground, under-inflated or worn tyre, etc.) represents a fundamental additional value for the optimal behavior of safety- and performance-oriented control logics. The non-linear model predictive control approach is employed to integrate the tyre varying dynamic parameters within the definition of physical constraints of the vehicle, guaranteeing the stability of the system and allowing to achieve the optimal solution for the defined vehicle instantaneous dynamic limits.

## 3. Physical Model, Physical Model-Based Control, and Virtual Scenario

To parametrize the vehicle and the tyres’ model, the authors have collected data with a chosen GT vehicle in a specific test session on track. Due to a non-disclosure agreement with the industrial research partner, the vehicle and the track will not be specified.

The track session has consisted of handling tests in the widest possible range of tyre operating conditions in terms of temperature, pressure, and wear level. Following the vehicle model parametrization and the tyre parameters’ estimation procedures described in [36,37], the vehicle non-linear system has been completely characterized in all the conditions of interest, being able to faithfully reproduce the experimental data in the virtual environment.

### 3.1. Vehicle Parametrization

The 14 degrees of freedom (DoF) vehicle model, based on the mathematical representation described in [38], has been modeled in a MATLAB/Simulink environment as follows:

- 6 DoF to reproduce longitudinal, lateral, vertical, pitch, roll, and yaw motion of the vehicle body;
- 4 DoF concerning the wheel rotation and 4 DoF for the wheel normal displacement, with the hypothesis that the degrees of freedom to the relative motion between the wheel and the vehicle body can be neglected along the longitudinal and lateral directions, allowing only the independent rotational and vertical displacements.

Furthermore, the parameterized vehicle is rear-wheel drive with front steering and internal combustion engine. The tyre model is described by Pacejka’s magic formula model, whose parameters have been characterized for different conditions of temperature, pressure, and wear. Per each road surface under study (dry, wet, snowy, and icy), the tyre-road friction coefficient has been supposed constant and is applied as an additional scaling factor of the $\lambda_{\mu_{x}}$ and $\lambda_{\mu_{y}}$ parameters [39], linearly combining the tyre characteristics identified on a reference road with the ones potentially achievable on diverse pavement surfaces.

**Figure 3:**
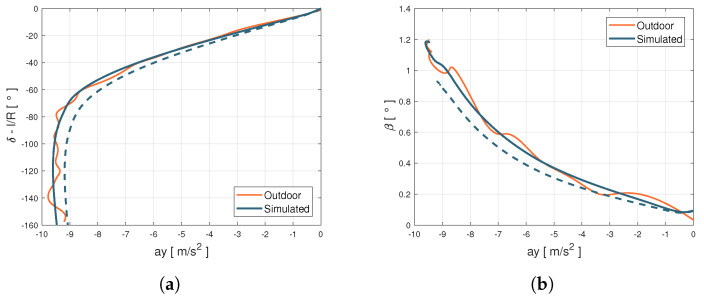
Comparison between outdoor acquisitions and simulation output. (**a**) Steering angle vs. lateral acceleration diagram. (**b**) Sideslip angle vs lateral acceleration diagram.

**Figure 4:**
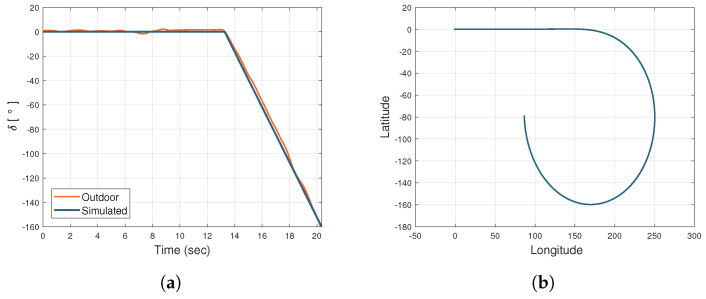
Example of lateral maneuver’s input reproduction. (**a**) Experimental and simulation steering angle comparison. (**b**) Slow-ramp-steer trajectory in virtual environment.

The vehicle dynamic behavior in the reference tyre conditions has been validated in a slow-ramp-steer maneuver, whose parameters are summarised in the Table 1 and outputs are illustrated in the Figure 3, feeding the model with the steering input presented in the Figure 4a):

For the validation purpose, lateral acceleration $a_{y}$, steering angle δ, side slip angle β have been compared for the same inputs. Figure 3 shows the comparisons between experimental data and model outputs shown on the classic $a_{y}-\delta$ and $a_{y}-\beta$ diagrams. An aspect that is worth pointing out is the difference between the black dashed and continuous lines: the first one is obtained using the starting parameters provided by the research partner, the second one is obtained employing the calibration procedure described in [37]. In particular, the starting under-steering characteristics (dashed lines) have been revised better identifying the parameters linked to the anti-roll bars stiffness and the steering maps, leading to a less under-steering behavior within the handling diagram, in agreement with the experimental data.

The enhanced parametrization has led to a higher slope in the linear section (Figure 3a), but also higher lateral grip and side-slip angle values, related to the rear axle behavior (Figure 3b). Once the vehicle and the tyres’ subsystems have been properly characterized in the specific range of temperature, pressure, and wear, the validity range of the MF tyre model has been extended adopting the MF-Evo one, described in [40]. In particular, the tyre model calibration process can be summarized in three fundamental steps: the first one is related to the pre-processing of the experimental data (which allows to discern useful information contained in the acquired data and to eliminate the non-physical outliers); the second one concerns the identification of the standard MF micro-coefficients in a specific range of temperature, pressure, and wear; the third step aims at the calibration of the additional multi-physical analytical formulations, taking into account of the entire dataset and, thus, extending the tyre model towards thermal and degradation phenomena.

**Figure 5:**
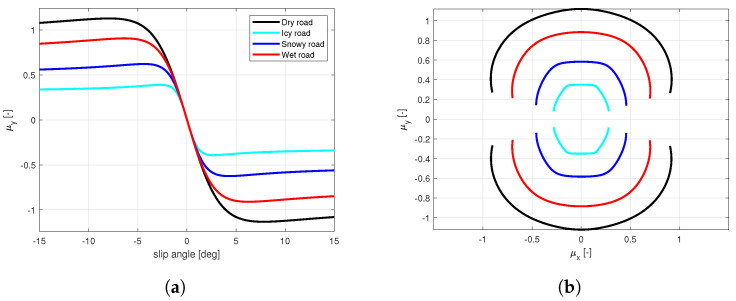
New tyre in optimal thermal condition in contact with different road surfaces. (**a**) Lateral interaction characteristics. (**b**) Adherence ellipse.

**Figure 6:**
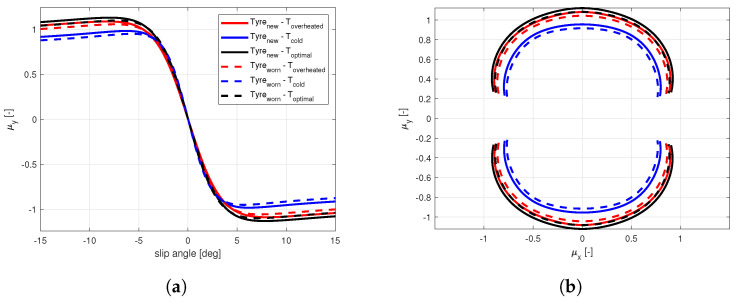
New and worn tyres in diverse thermal conditions in contact with the dry road. (**a**) Lateral interaction characteristics. (**b**) Adherence ellipse.

The calibration results are visible in terms of adherence ellipse in the Figure 1, where the experimental data have been compared towards the MF and MF-evo outputs within different temperature working ranges of the tyre. Finally, the parameters of the MF-evo model have been further modified to extend the applicability of the tyre model on different road surfaces, modifying the identified friction factors towards the pavement characteristics, as reported in the Table 2. The resulting interaction characteristics for different tyres, in diverse thermodynamic conditions and in contact with different road surfaces have been summarized in Figure 5 and Figure 6.

In steady-state conditions, the global force exerted by the tyres is in a dynamic equilibrium with the centrifugal force, as a function of the longitudinal velocity of the vehicle *v* and the instantaneous cornering radius *R*, relating the lateral acceleration $a_{y}$ and the longitudinal velocity *v* of the vehicle’s center of mass (CM) by the equation:

(1) $$a_{y}=\frac{v^{2}}{R};$$

**Figure 7:**
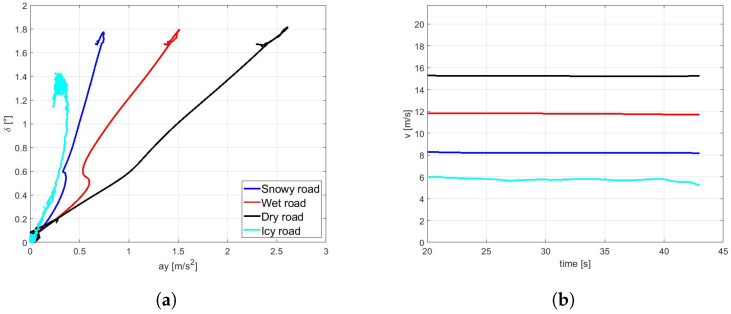
SRS maneuver on different road surfaces. (**a**) Vehicle understeer characteristics. (**b**) Maximum velocity achieved.

To demonstrate the potential influence of the road surface characteristics on the overall vehicle behavior, a set of simulations has been conducted with different tyre parameters described in Figure 5 in a steady-state lateral slow-ramp-steer (SRS) maneuver. The maximum achievable value of the forward velocity *v* for a given curvature and $a_{y}-\delta$ characteristics are reported for dry, wet, snowy, and icy pavement conditions in the Figure 7a,b, respectively.

### 3.2. Internal Vehicle Model

A four-wheel vehicle model based on the description in [16] has been used as the internal model for the NMPC controller. Specific characterization of load transfers, gear shift predictions, longitudinal force saturation, and an ellipsoidal tyre friction constraint have been also introduced in the model definition to improve the overall prediction capabilities of the controller. Finally, the model dynamics have been reformulated in spatial coordinates with respect to the curvilinear abscissa *s* along the track. In this way, track constraints can be defined with respect to space and the time can be considered as a minimization variable, as already highlighted in previous works [16,41,42].

**Figure 8:**
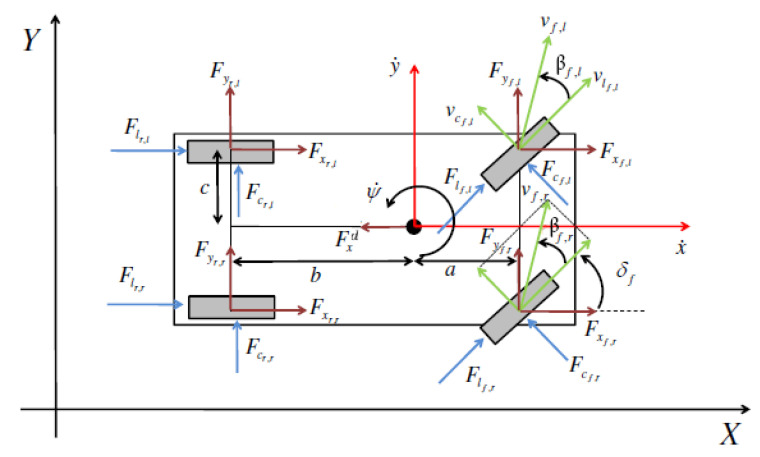
Internal vehicle model for control.

The continuous-time dynamics model is described as

(2) $$\dot{\xi }=\phi \left(\xi \left(t\right),u\left(t\right);p\left(t\right)\right),$$

where the state is represented by $\xi \left(t\right)\in R^{n_{x}}$, $u\left(t\right)\in R^{n_{u}}$ is the input, whereas the time-varying parameter vector is $p\left(t\right)\in R^{n_{p}}$.

(3) $$\begin{array}{cc} \ddot{x} & =\dot{y}\dot{\psi }+\frac{1}{m}\left(\sum_{i,j}F_{x_{i,j}}-F_{x}^{d}\right),\;\;\;\ddot{y}=-\dot{x}\dot{\psi }+\frac{1}{m}\left(\sum_{i,j}F_{y_{i,j}}\right),\\ \ddot{\psi } & =\frac{1}{I_{z}}\left[a\left(\sum_{j}F_{y_{f,j}}\right)-b\left(\sum_{j}F_{y_{r,j}}\right)+c\left(\sum_{i}F_{x_{i,r}}-\sum_{i}F_{x_{i,l}}\right)\right], \end{array}$$

where longitudinal and lateral positions are x,y, while ψ is the yaw angle. *m* and $I_{z}$ are the mass and the inertia around the vertical axis of the vehicle, respectively. a, b, c are the vehicle dimensional parameters, front wheels to CM longitudinal distance, rear wheels to CM longitudinal distance, and wheels to CM lateral distance, respectively. $F_{{\left\{x,y\right\}}_{\left\{i,j\right\}}}$ are the lateral and longitudinal forces on the wheels and $F_{x}^{d}$ is the longitudinal drag force in the vehicle’s reference frame. Subscripts i∈{f,r} refer to front or rear wheels, j∈{l,r} left or right wheels. Figure 8 illustrates the physical quantities involved and the reference systems chosen. $\delta_{f}$ is the steering angle of the front wheels, assumed the same for the both front tyres, and $\beta_{f,j}$ is the side slip angle of the f,j-th tyre. The projection of cornering and longitudinal forces in the vehicle frame, the position and the dynamics of the vehicle’s CM in the inertial frame X,Y, and the vehicle side slip angle β are described in [16], whereas the longitudinal drag force and the down-force are modeled as [43] pp. 97–98.

Differently from [16], the longitudinal tire forces in each wheel reference frame are computed as

(4) $$F_{l_{i,j}}=f_{{\mathrm{eng}}_{i,j}}-f_{{\mathrm{brk}}_{i,j}},$$

where the engine and braking forces are

(5) $$f_{{\mathrm{eng}}_{i,j}}=\mathrm{sat}\left(\frac{\tau_{{\mathrm{eng}}_{i}}}{r_{w}},\;\mu F_{z_{i,j}}\right),\;f_{{\mathrm{brk}}_{i,j}}=\mathrm{sat}\left(\frac{\tau_{{\mathrm{brk}}_{i}}}{r_{w}},\mu F_{z_{i,j}}\right),$$

where μ is the tyre friction coefficient, $r_{w}$ is the wheel radius and the saturation function is defined in (8). Then, the engine and braking torques at the wheels are:

(6) $$\tau_{{\mathrm{eng}}_{i}}=\gamma_{t}\;\left(\tau_{\mathrm{eng},i}^{\max}-\tau_{\mathrm{eng},i}^{\min}\right)+\tau_{\mathrm{eng},i}^{\min},\;\tau_{{\mathrm{brk}}_{i}}=\gamma_{b}\;\tau_{\mathrm{brk},i}^{\max},$$

where $\gamma_{t,b}$ are the normalized throttle and braking efforts, $\tau_{\mathrm{brk},i}^{\max}$ is the maximum torque given by the braking system to front/rear wheels, $\tau_{\mathrm{eng},i}^{\max}$ and $\tau_{\mathrm{eng},i}^{\min}$ are the maximum and minimum torque values expressed by the engine at front/rear wheels at a given gear and are changed as a time-varying parameter to the actual model gearshift. To compute the torques in the prediction horizon, an iterative strategy predicting the engine rpm, and, hence, gearshift, based on the predicted velocity is used [16]. Specifically, the engine rpm quantity is computed as

(7) $${\mathrm{rpm}}^{\mathrm{pred}}=\frac{v_{x}^{\mathrm{pred}}}{r_{w}}\frac{{\mathrm{diff}}_{\mathrm{ratio}}}{{\mathrm{gear}}_{\mathrm{ratio}}}\;\frac{60}{2\pi },$$

where ${\mathrm{diff}}_{\mathrm{ratio}}$ and ${\mathrm{gear}}_{\mathrm{ratio}}$ are the input/output torque ratios at the differential and at the gearbox (in a specific gear), respectively. The dependence of $\tau_{\mathrm{eng},i}^{\max,\min}$ w.r.t. the engine rotational velocity has been neglected. Finally, the saturation function is defined as:

(8) $$\mathrm{sat}\left(f_{a},f_{b}\right)=\frac{f_{b}}{1+\exp(-5\left(\frac{f_{a}}{f_{b}}-\frac{1}{2}\right))}.$$

The normal forces $F_{z_{i,j}}$ are modeled considering the load transfer in steady-state condition as described in [44]. The algebraic loop in the model has been avoided by considering $F_{x}^{\mathrm{sat}0}$ (total longitudinal force expressed in the vehicle frame saturated at nominal $F_{z}$) and $F_{y}^{\mathrm{static}}$ (the sum of the lateral forces computed at nominal $F_{z}$ on each wheel) as the forces used for the load transfer dynamics.

Finally, the lateral forces model is based on the simplified MF model described in [39] pp. 187–188, expressed by means of the macro-parameters B, C, D, E.

### 3.3. NMPC Algorithm

The goal of the NMPC controller for the virtual driver is to compute a reliable sequence of steering, throttle, brake commands in a prediction horizon, given a tailored cost function. The NMPC algorithm is based on MATMPC [10,45], an open source software built in MATLAB for real-time NMPC solution.

In MATMPC, a non-linear programming problem (NLP) is formulated at sampling instant *i* by applying direct multiple shooting [46] to an optimal control problem (OCP) over the prediction horizon $S=[s_{0},s_{f}]$, which is divided into *Nshooting intervals*$\left[s_{0},s_{1},\ldots ,s_{N}\right]$, as follows

(9) $$\min_{\xi_{\cdot |i},u_{\cdot |i}}\;\sum_{k=0}^{N-1}\frac{1}{2}{∥h_{k}\left(\xi_{k|i},u_{k|i}\right)∥}_{W}^{2}+\frac{1}{2}{∥h_{N}\left(\xi_{N|i}\right)∥}_{W_{N}}^{2}$$

(10) $$s.t.\;0=\xi_{0|i}-{\hat{\xi }}_{0},$$

(11) $$0=\xi_{k+1|i}-\phi_{k}\left(\xi_{k|i},u_{k|i};p_{k|i}\right),\;k\in \left[0,N-1\right],$$

(12) $${\underline{r}}_{k|i}\leq r_{k}\left(\xi_{k|i},u_{k|i}\right)\leq {\bar{r}}_{k|i},\;k\in \left[0,N-1\right],$$

(13) $${\underline{r}}_{N|i}\leq r_{N}\left(\xi_{N|i}\right)\leq {\bar{r}}_{N|i}$$

where $\xi_{\cdot |i}={\left(\xi_{0|i}^{⊤},\xi_{1|i}^{⊤},\ldots ,\xi_{N|i}^{⊤}\right)}^{⊤}$, and $u_{\cdot |i}={\left(u_{0|i}^{⊤},u_{1|i}^{⊤},\ldots ,u_{N-1|i}^{⊤}\right)}^{⊤}$, while ${\hat{\xi }}_{0}$ represents the measurement of the current state. System states $\xi_{k|i}\in R^{n_{\xi }}$ are defined at the discrete arc-length point $s_{k}$ for k=0,…,N and the control inputs $u_{k|i}\in R^{n_{u}}$ for k=0,…,N−1 are piece-wise constant. Their definitions are given in (14) and (15). Here, (12) is defined by $r\left(\xi_{k|i},u_{k|i}\right):R^{n_{\xi }}\times R^{n_{u}}\to R^{n_{r}}$ and $r\left(\xi_{N|i}\right):R^{n_{\xi }}\to R^{n_{l}}$ with lower and upper bound ${\underline{r}}_{k|i},{\bar{r}}_{k|i}$. Equation (11) refers to the *continuity constraint* where $\phi_{k}\left(\xi_{k|i},u_{k|i};p_{k|i}\right)$ is a numerical integration operator that solves (16) with initial condition $\xi \left(0\right)=\xi_{0|i}$ and returns the solution at $s_{k+1}$. The time has been included as a state variable with the following ODE $\dot{t}=\frac{1}{\dot{s}}$ to fulfil the minimization of the travel time over the prediction horizon. The full state vector is then given by:

(14) $$\xi ={\left[\dot{x},\dot{y},\dot{\psi },e_{\psi },\;e_{y},\;\delta_{f},\;\gamma_{t},\;\gamma_{b},t\right]}^{T},$$

where $e_{\psi }$, $e_{y}$ are orientation and lateral error of the vehicle with respect to the center-line of the path, respectively. The input computed by the algorithm is then:

(15) $$u={\left[{\dot{\delta }}_{f},\;\dot{\gamma_{t}},\;\dot{\gamma_{b}},\;\epsilon_{\mathrm{slip}},\;\epsilon_{\mathrm{err}},\;\epsilon_{\mathrm{gg}}\right]}^{T},$$

where ${\dot{\delta }}_{f},\;\dot{\gamma_{t}},\;\dot{\gamma_{b}}$ are the derivatives of the actual input to the vehicle and ϵ are slack variables. This formulation allows a smooth action of the controller and avoids too aggressive, unrealistic behaviors.

The dynamics equation of the model used in the NMPC algorithm can be compactly written as

(16) $$\xi^{\prime }=\phi \left(\xi \left(s\right),u\left(s\right);p\left(s\right)\right),$$

where $p\left(s\right)={\left[\zeta \left(s\right),\tau_{\mathrm{eng},i}^{\mathrm{MAX},\min}\left(s\right)\right]}^{T}$.

The real-time iteration scheme (RTI) [47] is employed to reduce the time required to solve the (9) problem. Moreover, a non-uniform grid strategy [48] has been used for lowering the computational burden and let the controller predict a sufficiently long horizon (chosen 400 m in advance for the specific vehicle).

The cost function for the NMPC is defined as:

(17) $$\begin{array}{cc} h_{k}\left(\xi_{k},u_{k}\right)= & [\beta ,\gamma_{t}\cdot \gamma_{b},\zeta \cdot \gamma_{t},t,\dot{\delta_{f}},{\dot{\gamma }}_{t},{\dot{\gamma }}_{b},\epsilon_{\mathrm{slip}},\epsilon_{\mathrm{err}},\epsilon_{\mathrm{gg}}{]}^{⊤},\\ h_{N}\left(\xi_{N}\right)= & [\beta ,\gamma_{t}\cdot \gamma_{b},\zeta \cdot \gamma_{t},t,e_{y}-e_{y}^{\mathrm{ref}},{\dot{e}}_{y},e_{\psi }-e_{\psi }^{\mathrm{ref}}+\\ & +\beta ,{\dot{e}}_{\psi }{]}^{⊤}. \end{array}$$

The penalty on the vehicle side slip β is used to limit the sliding behavior of the vehicle; simultaneous throttling and braking are penalized by the cost $\gamma_{t}\cdot \gamma_{b}$. The $\zeta \cdot \gamma_{t}$ cost is included to make the controller accelerate smoothly during the final phase of the track corner exit. The objective variable time *t* is added to minimize the time on the prediction horizon. Smooth control actions are ensured by the objective terms on the inputs. The three slack variables are also adopted to define the *soft constraints* [49], which increase the robustness of the overall procedure. Finally, the terms related to errors $e_{y}\;\mathrm{and}\;e_{\psi }$, used only as terminal objective variables, are introduced to integrate information about the trajectory over the prediction horizon.

The constraints are defined as

(18) $$\begin{array}{cc} r_{k}= & [\delta_{f},\gamma_{t},\gamma_{b},{\dot{\delta }}_{f},\dot{\gamma_{t}},\dot{\gamma_{b}},\epsilon_{\mathrm{slip}},\epsilon_{\mathrm{err}},\epsilon_{\mathrm{gg}},\beta +\epsilon_{\mathrm{slip}},\\ \; & e_{y}+\epsilon_{\mathrm{err}},{\left(\mu_{x}\frac{{\ddot{x}}_{\mathrm{ext}}}{g}\right)}^{2}+{\left(\mu_{y}\frac{{\ddot{y}}_{\mathrm{ext}}}{g}\right)}^{2}+\epsilon_{\mathrm{gg}}{]}^{⊤},\\ r_{N}= & [\delta_{f},\gamma_{t},\gamma_{b}{]}^{⊤}, \end{array}$$

where the constraints on $\delta_{f},\gamma_{t},\;\mathrm{and}\;\gamma_{b}$ are intrinsic bounds of the actual vehicle controls, while those on ${\dot{\delta }}_{f},{\dot{\gamma }}_{t},\;\mathrm{and}\;{\dot{\gamma }}_{b}$ are added in order to improve the smoothness of the computed inputs and can be used to easily tune the aggressivity of the NMPC driving commands. Additionally, the slack variables have been constrained in order to help the optimization procedure restricting the *search space* of the inputs. The slack variables are used for defining the soft constraints: the first one is introduced on the side-slip of the vehicle and helps the controller to regain control of the vehicle in case of high skidding; the second one is used to correct the trajectory when the vehicle is out of track; the third one instead is designed to make the controller respect the required *gg diagram*, which represent the maximum combined longitudinal-lateral acceleration that can be induced by the combined longitudinal-lateral behavior of the specific tyre spec [50]. $\mu_{x}$ and $\mu_{y}$ are the longitudinal and lateral friction coefficient of the tyres, respectively, whereas the considered accelerations on the vehicle are

(19) $$\begin{array}{cc} \; & {\ddot{x}}_{\mathrm{ext}}=\frac{\sum_{i,j}F_{x_{i,j}-F_{d}^{x}}}{m},\\ \; & {\ddot{y}}_{\mathrm{ext}}=\frac{\sum_{i,j}F_{y_{i,j}}}{m}. \end{array}$$

At the *i*-th sampling instant, considering that the QP solution is $\Delta u^{i^{*}}$, the control input is updated by

(20) $$u^{i^{*}}=u^{i-1^{*}}+\Delta u^{i^{*}},$$

The first sample of $u^{i^{*}}$ is applied to the vehicle, the prediction horizon is shifted forward and the optimization procedure is repeated with updated state measurement.

### 3.4. Co-Simulation Environment

**Figure 9:**
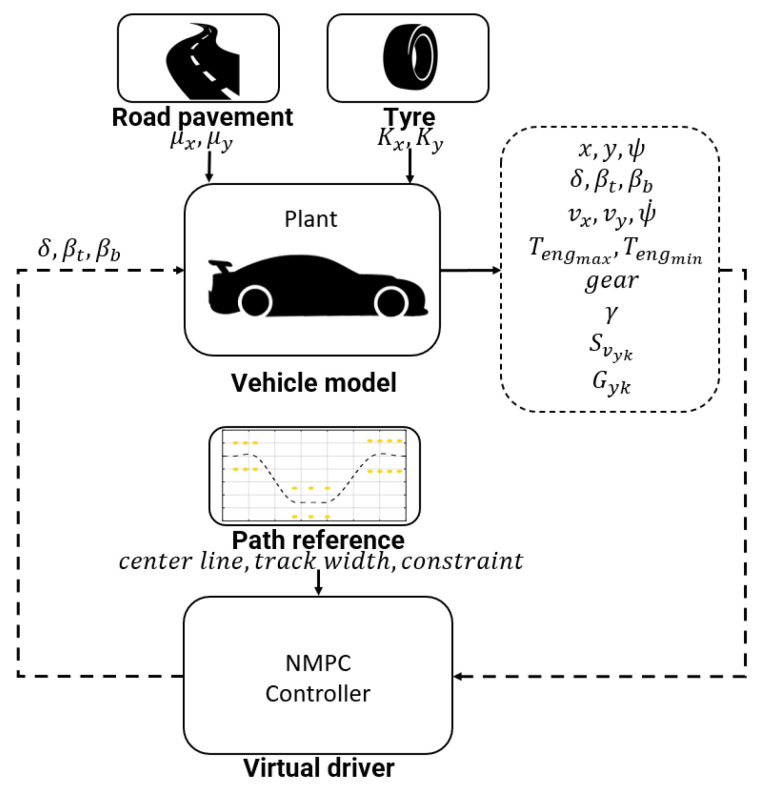
Co-simulaton platform.

The co-simulation platform, represented in the Figure 9, is composed of the following subsystems:

- Plant model: a 14 DoF vehicle model reproducing the overall vehicle dynamics behavior;
- Road pavement: a boundary condition module concerning the asphalt condition and computing the tire-road friction coefficient to reproduce dry, wet, snowy, and icy contact;
- Tyre: MF-Evo tyre model reproducing the tyre dynamic behavior in different thermal and wear conditions;
- Path reference: the track geometrical representation defined by the specific maneuver and employed to compute the cost function.

The maneuver chosen for the current study is the emergency double-lane-change maneuver, generally performed on the highway to overtake another vehicle [51]. The test is commonly adopted because it correlates the ability of controlling the vehicle at the limits of handling with an enhanced safety for the vehicle occupants in scenarios concerning the presence of obstacles on the path [52]. Given the criteria for ideal lane-change path, prescribing a minimal length path with a smooth and continuous curvature at a given vehicle forward velocity, the trajectory of the DLC maneuver is computed without violating the track boundaries and assuring that all the tyres remain always in contact with the road surface (possible lift motions are avoided with constraints modelled within the maximum load transfers, as described in [9]).

The co-simulation is conducted in MATLAB/Simulink environment, coupling the plant model with NMPC controller and performing the dynamic simulation of the plant model at $f_{sim}$ = 1000 Hz, while the control action is updated by NMPC at $f_{ctrl}$ = 100 Hz. The simulations have been computed on a Windows 10 machine with Intel(R) Core(TM) i7-7700HQ @ 2.80GHz CPU.

## 4. Analysis and Results

The knowledge of the instantaneous and potential grip directly on board and in realtime potentially allows the vehicle control logic to maximize the probability of avoiding obstacles and reducing the severity of collisions. To investigate the possible outcomes of a model-based control within a vehicle safety-linked scenario, the authors have performed within the DLC maneuver a complete design of experiment comprehending:

- *Case A:* the adoption of two different sets of NMPC weights (best and global) in the definition of the cost function.
  The best NMPC set of weights addresses the maximum achievable performance of the underlying vehicle plant model, specifically calibrated for a new tyre working in the optimal thermal range in contact with the dry road, whereas the global NMPC set of weights represents the trade-off solution to guarantee ability of the vehicle to complete the DLC maneuver in the worst proposed dynamic scenario, i.e., a worn cold tyre in contact the icy road surface. In this case, the parameters of the plant and the controller models are the same for each simulation;
- *Case B:* the analysis of the vehicle dynamic response in case of different tyre thermal and ageing conditions on the same road and in case of the tyre with a specific thermal and wear state on different pavements. In this case, the parameters of the plant and the controller models are the same for each simulation;
- *Case C:* the possibility to employ the non-linear model predictive controller calibrated with the average set of weights in conditions where the parameters of the controller model can be updated in real-time on the basis of the actual state of the plant model or can be constant and with an estimation on the friction value affected by a percentage error respect the real value.
  This particular scenario has been conducted to highlight the importance of the correct estimation of the parameters of the controller model, potentially aware of the actual knowledge of tyre-road friction. The simulation outputs with average tyre parameters within the controller model have been compared towards the ones obtained with the instantaneous parameters of the co-simulated vehicle plant to put in evidence the importance of the correct information concerning the tyre friction and stiffness for the vehicle dynamics control.

The simulation outputs have been compared in terms of the vehicle trajectory, the forward velocity, the vehicle side slip angle, and yaw angle.

### 4.1. *Case A*

In this section, the impact of two possible sets of weights, defined within the NMPC cost function, is investigated. Both the plant and controller models share the same model parameters of a new tyre in the optimal thermal window in contact with the dry road.

The *best* set of NMPC weights represents the most suitable solution to perform the DLC maneuver with both the plant model and the controller model in the maximum performance conditions of the tyre, corresponding the the maximum dynamic limits of the vehicle. The *global* set of NMPC weights stands for the conservative trade-off solution, calibrated to guarantee the accomplishment of the maneuver in all the possible tyre-linked and boundary conditions, in which the plant and controller models share the same physical parameters (i.e., the performance of the vehicle controller is limited by the worst possible dynamic scenario of a cold and worn tyre in contact with the icy road).

**Figure 10:**
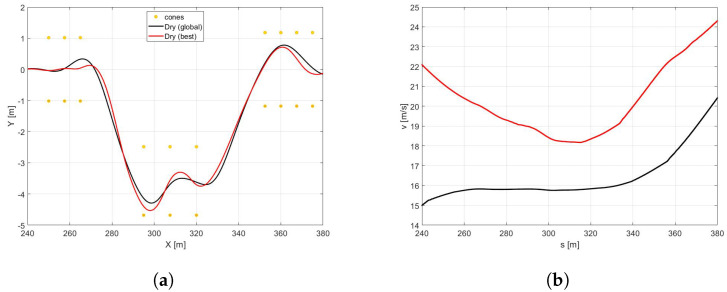
(**a**) Vehicle trajectory performed in the DLC maneuvers in a different road surface (dry in black, wet in red, snow in blue, and icy in light blue), but with the same tyre (new tyre in optimal range temperature) for a NMPC tuned to better perform the maneuver in all road surface, tyre, and temperature condition. (**b**) Vehicle velocity.

**Figure 11:**
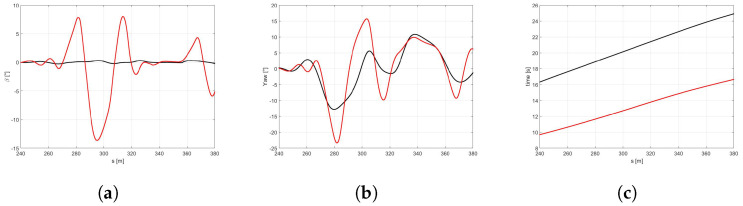
(**a**) β angle. (**b**) Yaw angle. (**c**) Time.

In the Figure 10a the trajectories of the vehicle with the *best* (red) and *global* (black) sets of NMPC weights are compared. It is easy to observe that the optimized set of weights allows the vehicle performing at a larger trajectory and achieving significantly higher velocities both in the first part of the curves and at the end of the DLC maneuver (Figure 10b). It is worth highlighting that the *best* set also demonstrates higher side slip and yaw angles (Figure 11a,b), because it is specifically optimized to perform in the scenario of a new optimal tyre in contact with the dry road, therefore allowing the vehicle to reach the actual friction limits. Furthermore, the *best* set allows the vehicle to approach to the DLC manuever and to end the scenario 6.62 and 8.34 s before, respectively (Figure 11c).

### 4.2. *Case B*

In this section, only the *global* set of NMPC weights has been employed to compare the dynamic response of the vehicle in two scenarios: (1) different road characteristics (dry, wet, snowy, and icy) with the new tyre within the optimal thermal range, and (2) different tyre thermal and ageing conditions in contact with the dry road. The plant and the controller models share the same physical parameters for each iteration.

**Figure 12:**
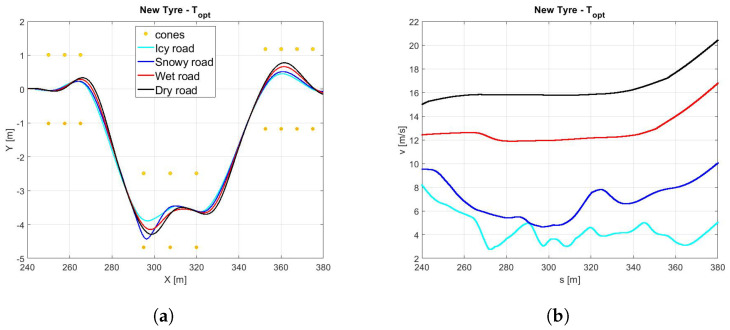
(**a**) Vehicle trajectory. (**b**) Vehicle velocity.

**Figure 13:**
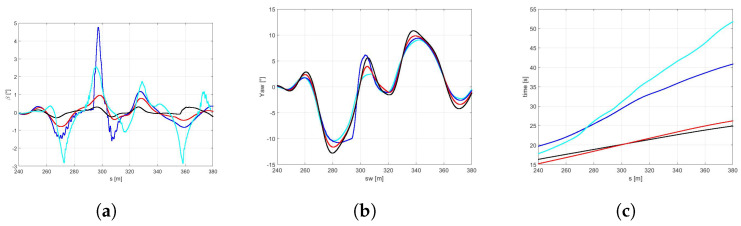
(**a**) Side slip angle. (**b**) Yaw angle. (**c**) Time.

**Figure 14:**
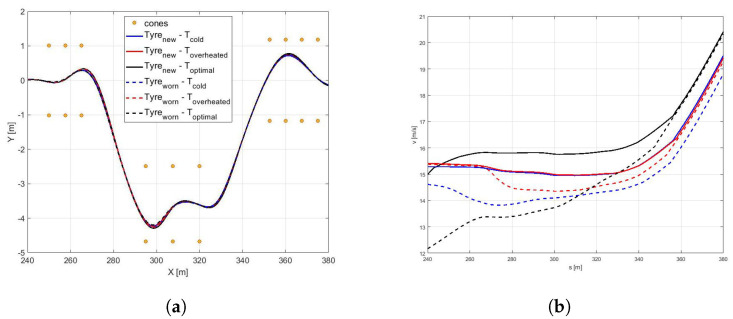
(**a**) Vehicle trajectory performed in the DLC maneuvers in a dry road, with different tyre condition (New tyre (continuous lines) and worn tyre (dashed lines) in optimal (black), cold (blue), and overheated (red) temperature range. (**b**) Vehicle velocity.

- *Scenario B1*
  In the Figure 12a it is possible to observe how the vehicle maneuver characterized by the highest friction coefficient (dry pavement) performs the DLC with a largest trajectory and the highest velocity Figure 12b in minimum amount of time Figure 13c and Table 3.
  Since the *global* NMPC set is limited by the most critical dynamic condition (worn cold tyre in contact with the icy road), the Figure 13a shows higher values in terms of side slip angle for snowy and icy road surfaces, foreseeing the possibility to perform the maneuver in more aggressive way for dry and wet road conditions.
  Such a conservative behavior can be motivated by the fact that the *global* set of weights is a result of a trade-off between completely different dynamic scenarios in the respect of vehicle maneuverability and safety.
- *Scenario B2*
  The comparison between a same road condition (dry) performing with different tyre condition (new or worn, in the optimal temperature range, cold or overheated) are shown in the following figure. Regarding the analysis of trajectories, shown in the Figure 14a it is possible to observe how they are too similar each other due to the same road pavement, however in the new tyre condition a little largest trajectory has been carried out to achieve an highest velocity Figure 14.

**Figure 15:**
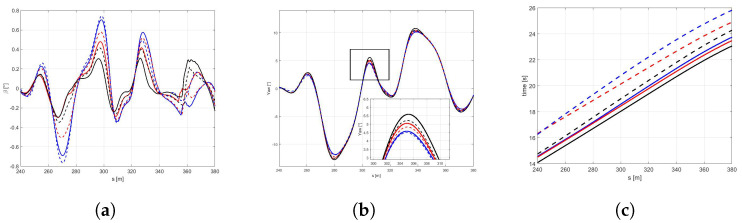
(**a**) Side slip angle. (**b**) Yaw angle. (**c**) Time.

The analysis side slip angle show a dependence of β angle with the tyre stiffness, indeed the highest value of β has been performed to highest cornering stiffness Figure 15a. Finally, in the Table 4 are shown the performing time for each condition.

### 4.3. *Case C*

**Figure 16:**
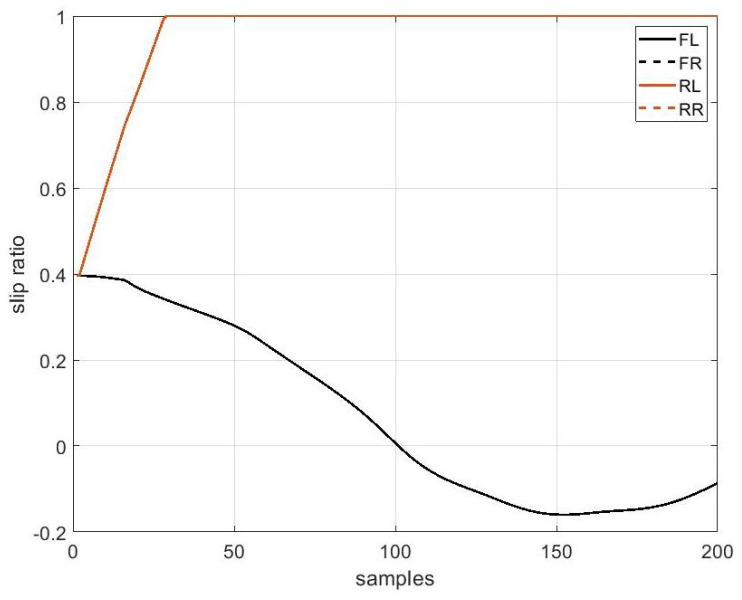
Slip ratio achieved for the four tyres.

**Figure 17:**
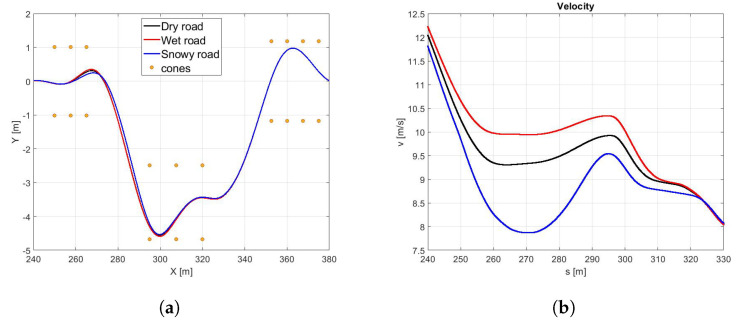
(**a**) Vehicle trajectory performed in the DLC maneuvers in a dry, wet, and snow road, with new tyre in optimal range temperature. (**b**) Vehicle velocity.

**Figure 18:**
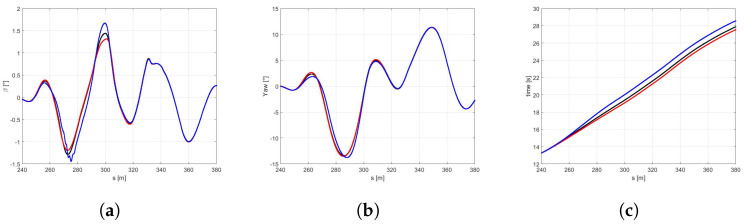
(**a**) Side slip angle. (**b**) Yaw angle. (**c**) Time.

- *Scenario C1*
  In this paragraph the aim of the authors is to argue the following query:
*If the plant and the controller do not share the same model parameters, i.e., the parameters of the controller model are not updated by a specific co-simulated estimator of the vehicle parameters and state, and of the tyres’ and the road conditions are not known a priori, how a controller model with an average "parameters’" configuration could perform with different plant model employment scenarios within the DLC maneuver?*
  With this purpose, the controller model has been fed with the parameters of friction and stiffness corresponding the mean value of the all possible tyre-road conditions explored.
  It is worth highlighting that, as expected, it is not possible to perform the DLC maneuver with the icy road with the above configuration. Indeed, as appears clear in the Figure 16, the rear axle achieves the maximum slip ratio, not allowing to complete the simulation in safety.
  For this reason, in the following figures, only dry, wet, and snowy road conditions are reported. In the Figure 17a,b it is possible to observe how the difference between the three pavement surfaces are less pronounced towards the results discussed in Scenario B. Moreover, the vehicle in contact with the wet road achieves a maximum velocity, even higher than with the dry surface, completing the maneuver in less time Figure 18c).
  The reason for such behavior can be conducted to the conservative control action, particularly visible in dry boundary condition, since the absolute difference in terms of the friction limit is particularly high between the plant and the controller models in this scenario. Indeed, in the Figure 18a the the side slip angle is similar for three conditions explored. Furthermore, even the maneuver in snow conditions is achieved in a comparable time period, since the friction limit of the average controller model is similar to the one of the plant model working in snowy boundary conditions Figure 18b.
- *Scenario C2*
  Remarking that an accurate online friction coefficient estimation becomes absolutely necessary to allow exploiting the vehicle dynamics in maximum performance conditions within a combined DLC maneuver, in this paragraph the aim of the authors is to argue another possible query: *In a real scenario, where an onboard tyre-road friction estimator able to estimate (among others) the grip parameter with a certain degree of accuracy and to update the control model parameters in run-time, is available, how a controller model with a percentage error concerning the vehicle instantaneous conditions could perform within the same maneuver?*
  With this purpose, the parameter concerning the tyres’ friction of the controller model has been considered with an intrinsic error with a supposed standard deviation of ±15% respect to the actual grip value of the vehicle plant model.

**Figure 19:**
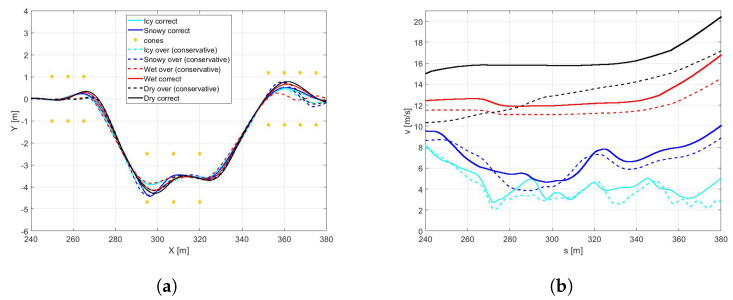
(**a**) Vehicle trajectory performed in the DLC maneuvers in conservative vs global configuration. (**b**) Vehicle velocities.

**Figure 20:**
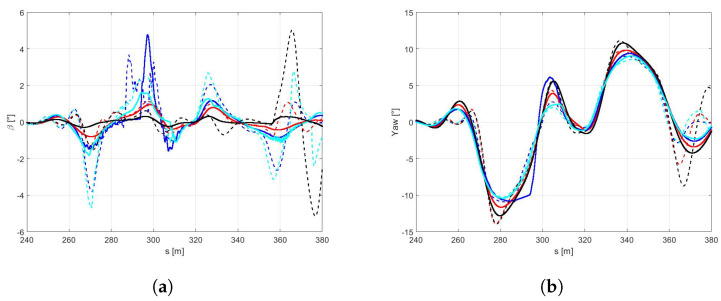
(**a**) Side slip angles. (**b**) Yaw angles.

It is worth noting that in a scenario where the grip factor is overestimated, the controller with the global configuration of the cost function computes more aggressive control actions leading to out-of cones trajectories and undesirable sliding effect. To avoid this issue, a robust global configuration has been introduced in order to let the controller being effective in managing the vehicle behavior in overestimated grip-linked scenarios. The above new configuration leads to more conservative actions and, consequently, to a considerable loss of performance in terms of velocity. In particular, the loss in performance in terms of the average speed (in percentage) in the four cases analyzed has been objectively quantified in Table 5. In Figure 19 and Figure 20, the performance obtained by the two configurations in terms of trajectories, speed, side-slips, and yaw angles are also compared. Notably, the side-slip in Figure 5 reaches peaks of 5 degrees, confirming that the configurations obtained controls the vehicle at the limit of handling.

## 5. Conclusions

The global autonomous mobility industry, growing at a rapid pace, is an intrinsically multidisciplinary field that aims at designing advanced onboard control logics by integrating principles from different disciplines including mechanical, control, and computer science engineering, legal, social, and economic fields. The multidisciplinary proposal will provide a paradigm shift in the development of strategies and a solid breakthrough towards enhanced development of the driving automatization systems.

In the proposed work, the authors have investigated how accurate information regarding the state of the real system of the parameters concerning the controller model could affect the behavior of the real system, represented in the form of the high-fidelity validated plant model. The influence of the tyre thermal dynamics, the impact of the possible ageing effects and the contact with different road pavements have been examined. Wrong parameters in the definition of the internal model of the NMPC might compromise the control performance, especially when the vehicle is supposed to drive at the limit of handling conditions. Specifically, a controller characterized by an overestimation of the grip conditions is forced to compute too aggressive control actions that might bring the vehicle in unstable and unsafe conditions, that are very difficult to handle for the controller itself. On the contrary, an underestimation of the grip might reduce the performance of the controller, which is forced to compute too conservative control actions. Moreover, the parameters of the cost function play an important role in defining the level of performance that the controller is required to achieve. A high weight on the travel time forces the vehicle to drive fast along the path, hence requiring effective proper internal model parameters to describe the vehicle behavior at the limit of handling. Instead, a more conservative tuning (i.e., high weights on side-slip, lateral error, orientation error) can be effective with also less precise coefficients, as the vehicle is not supposed to travel at the limit of handling. Due to these statements, in future work, the effects of including a real-time estimate of the tyre and the environment states, along with an adapting strategy for the weights of the cost function will be included in the whole analysis.

The investigation constitutes a part of the broader panorama of studies on novel model-based ADAS systems, which could become adaptive to the system state and boundary conditions, by means of a real-time physical model-based estimator of adherence, sensitive to environmental conditions and to the instantaneous state of tyres. In this way, the physical limits of the real system could become totally exploited by the control logic, minimizing the safety-related risks, and unleashing the potential of physical modeling to the next level of vehicle control.

## Figures and Tables

**Table 1:** Slow-ramp-steer inputs.

Description	Value	Unit
start time	13.26	s
end time	20.3	s
initial velocity	27.9	m/s
initial gear	3	-
ramp duration	7.04	s
initial steer	0	deg
slope steer	−22.29	deg/s

**Table 2:** Summary of the velocity maximum values assumed for each road scenario.

Friction Coefficient μ	Lateral Acceleration $a_{y}$	Longitudinal Velocity *v*
[−]	[m/s${}^{2}$]	[m/s]
0.35	0.35	5.92
0.55	0.70	8.37
0.80	1.50	12.2
1.00	2.52	15.9

**Table 3:** Summary of time’s maneuver for each scenario.

Road Surface	Time [s]
Dry	24.8
Wet	26.2
Snowy	40.8
Icy	51.7

**Table 4:** Summary of time’s maneuver for each scenario.

Tyre Condition	Time *s*
$New-T_{opt}$	23.0
$New-T_{cold}$	23.7
$New-T_{overheated}$	23.44
$Worn-T_{opt}$	24.24
$Worn-T_{cold}$	25.8
$Worn-T_{overheated}$	24.8

**Table 5:** Summary of the difference in velocity mean values (%) and lateral error assumed for each road scenario.

Road Surface	Friction Estimation	Longitudinal Velocity *v*
[−]	[−]	[%]
Icy	correct	−
Icy	overestimation	−16.02
Snowy	correct	−
Snowy	overestimation	−8.36
Wet	correct	−
Wet	overestimation	−8.30
Dry	correct	−
Dry	overestimation	−21.00

## Data Availability

Data available on request due to industrial confidential agreements.
